# North American Football Fans Show Neurofunctional Differences in Response to Violence: Implications for Public Health and Policy

**DOI:** 10.3389/fpubh.2018.00177

**Published:** 2018-07-06

**Authors:** Thomas A. Daniel, Kyle M. Townsend, Yun Wang, David S. Martin, Jeffrey S. Katz, Gopikrishna Deshpande

**Affiliations:** ^1^AU MRI Research Center, Department of Electrical and Computer Engineering, Auburn University, Auburn, AL, United States; ^2^Department of Psychology, Westfield State University, Westfield, MA, United States; ^3^Department of Psychology, Auburn University, Auburn, AL, United States; ^4^Cecil B. Day School of Hospitality Administration, Robinson College of Business, Georgia State University, Atlanta, GA, United States; ^5^Department of Psychiatry, Columbia University, New York, NY, United States; ^6^Department of Nutrition, Dietetics, and Hospitality Management, Auburn University, Auburn, AL, United States; ^7^Center for Neuroscience, Auburn University, Auburn, AL, United States; ^8^Center for Health Ecology and Equity Research, Auburn University, Auburn, AL, United States

**Keywords:** North American football, contact sports, violence, empathy, functional MRI

## Abstract

While social and behavioral effects of violence in the media have been studied extensively, much less is known about how sports affect perceptions of violence. The current study examined neurofunctional differences between fans and non-fans of North American football (a contact sport) while viewing violent imagery. Participants viewed images of violence in both football and non-football settings while high-resolution functional magnetic resonance imaging (fMRI) data were acquired from their brains. Neurological activation was compared between these violence types and between groups. Fans of football show diminished activation in brain regions involved in pain perception and empathy such as the anterior cingulate cortex, fusiform gyrus, insula, and temporal pole when viewing violence in the context of football compared to more broadly violent images. Non-fans of football showed no such effect for the types of violent imagery and had higher activation levels than fans of football for the specified brain regions. These differences show that fans of football may perceive violence differently when it is in the context of football. These fan attitudes have potential policy implications for addressing the issue of concussions in North American football.

## Introduction

Repeated exposure to violence alters both behavioral and neurological responses toward violent materials in otherwise healthy individuals. For example, frequent exposure to violence, such as that in video games or movies, may lead to the habituation of violent behaviors (1) and more pro-violent attitudes (2). While causal mechanisms underlying these changes are still debated (3), Anderson and Bushman's (1) General Aggression Model provides several testable predictions regarding violent media consumption. According to this model, violence in the media serves as a model for behavior through observational learning, creates schemas using aggressive behaviors, and reduces emotional responsiveness toward violence. This last factor serves to reduce empathy in those that are exposed to violence (12). By studying these separate mechanisms and their neurofunctional basis, future violent behaviors can be reduced or prevented (4). Therefore, this topic is of clear relevance to public health policy. Studies exploring the effects of violence on emotion and attitudes have historically focused on television (primarily violence in movies) and video games. Less is known about how other violent media, such as watching contact sports (e.g., North American football), may affect behavior and attitudes.

The attitudes of individuals watching the game impacts the violence experienced by those playing the game. Concerning North American football, it is thought that repetitive brain trauma in players is an etiological factor in the development of chronic traumatic encephalopathy, neurodegeneration, depression and suicidal risk (5). This brain trauma is an issue for professional athletes and youth football players; who may also be exposed to large numbers of repetitive head impacts (6). Therefore, concussions and sub-concussive blows to the head commonly found in North American football can be considered to be an urgent public health burden which requires a policy response either from the government or the sporting body. However, making rule changes in this sport can be difficult as the attitudes of many fans and players oppose any such changes (7). Additionally, previous research has indicated that fans gain more pleasure and excitement when viewing non-scripted violence (which is a cornerstone of North American football) than when they watch sports without violent interactions (8–10). This effect may also help to explain why fans are resistant to rule changes that increase player safety but also reduce the amount of violence during play. We surmise that this resistance may be driven by repeated exposure of fans to violence in this sport, with concomitant neural and behavioral manifestations. Below, we provide a background on behavioral and neural literature on empathy and violence which will then provide a basis for a more specific hypothesis derived from the viewpoints expressed above.

Empathy serves as a way for one individual to take the perspective of another, either affectively or cognitively (11). This ability to take another's perspective predicts the usage of prosocial behaviors such as sharing and helping. Funk et al. found increased exposure to media violence predicted lower rates of empathy. As the General Aggression Model predicts, these low rates of empathy were also associated with pro-violent attitudes. Examples of pro-violent attitudes include beliefs that weapons such as knives or guns are fashionable or that it is acceptable for parents to let their children fight (2). To further explore this connection, Krahe and Moller (12) tracked adolescents for over a year, recording their violent media usage, aggression, and empathy. Their findings confirm those of Funk et al. but the longitudinal design offers a clearer view of the causal mechanisms. This connection between increased violent media consumption contributing to lower levels of empathy was again replicated by Mößle et al. (13), showing that empathy fully mediated aggressive behaviors.

The connection between violence and empathy has also been explored using neuroimaging techniques. Using fMRI, Guo et al. (14) demonstrated neurofunctional differences in individuals exposed to violent videos. Participants viewed a short video of either violent or non-violent nature, followed by a set of images depicting painful bodily harm. Participants that viewed the violent video beforehand showed reduced activation in the anterior mid-cingulate cortex (aMCC) and anterior insula (AI), two areas commonly found to be active while encoding emotionality and pain (15, 16). These results highlight short-term effects of violence exposure and empathy in the brain, but other studies have examined the long-term effects. The same kind of exposure to violence has been shown to alter functional connectivity in brain networks such as the default mode network, reward network (17) and fronto-parietal network (18).

Bartholow et al. (19) using EEG, grouped individuals by video game consumption, creating non-violent game players and violent game players. Participants viewed negatively valenced images of both violent and nonviolent nature. Participants that frequently played violent games showed a reduced P300 amplitude while viewing violent images compared to the group that played nonviolent games. Guo et al. (14) and Bartholow et al. (19) attribute these findings as causal; individuals exposed to violence show neurofunctional differences that may cause desensitization toward violent images and decreases in empathy.

Here, we examined how exposure to North American football alters the neural response to violence. This is an essential examination for multiple stakeholders of North American football. Sporting bodies are interested in both making the game safer for players, as much focus has fallen on the potential adverse effects of North American football, as well as ensuring fan satisfaction with the product on the field. Many fans have opposed rule changes that promote player safety as they see this as changing the game they enjoy (7). Thus North American football finds itself caught in an unenviable position, with certain segments of society calling for rule changes to increase players' safety while fans, who help fund the multibillion-dollar industry that is North American football, may view violence differently than the individuals calling for change. Therefore, gaining a better understanding of the neural mechanisms of fans (and how they differ from non-fans) is vital to better inform the policies and overall governance of North American football.

According to the General Aggression Model (1), we predicted that repeated exposure to North American football, a violent contact sport, would reduce the neural activation of areas responsible for emotion regulation, perception of others' pain and empathy [e.g., in regions such as the amygdala (20), anterior cingulate (21, 22)]. To test this hypothesis, individuals were recruited and grouped based on their history of consuming football-related entertainment. While in a high-resolution 7T MRI scanner, participants viewed two types of violence images: images of general violence (e.g., someone being struck with a fist) and images of football-related violence (e.g., a rough tackle). Brain activation during this task was measured and compared across the two groups (football fans vs. non-football fans) and between condition (general violence vs. football violence).

## Materials and methods

### Participants

This study was approved by and carried out in accordance with the recommendations of Auburn University Institutional Review Board. All subjects gave written informed consent in accordance with the Declaration of Helsinki. The experiment was conducted at the Auburn University MRI Research Center.

Fifteen right-handed, college-educated participants (53% female; 60% Caucasian; ages 22–57, *M* = 30.7, *SD* = 10) were recruited from the Auburn, Alabama area. 94% of the participants were currently employed, and all of the participants had at a minimum a bachelor's degree. Participants were pre-screened to determine their status as a football fan using a “Team Identity Scale” and their ability to take part in an MRI scan. Team identity was measured utilizing a modified version of the seven items found in the Sport Spectator Identification Scale (23). The modification of the scale was made in the actual scaling, which was changed from an 8 point Likert type scale to a 5 point Likert type scale based on feedback from focus groups in a previous study that also incorporated the same measure (24). Higher scores indicated that the person was an identified fan of the team for which he or she was rooting. We averaged the seven items (the questions that were part of the survey are provided as supplementary material) to form our team identity variable. Further, individuals who reported pre-existing medical conditions, claustrophobia, or ferrous metals in their body were excluded.

Two groups were formed based upon participants' self-disclosure as a North American football fan, at both the national and college level. Participants were grouped as Fans (*N* = 7) or Non-Fans (*N* = 8) based on their response in the Team Identity Scale (23), using a cutoff score of 4.5, when all item responses were averaged.

### Stimulus development

Stimuli related to general violence were selected from the International Affective Picture System [IAPS; (25)], a library of normative emotional images for experimental investigations of emotion and attention. Images in the IAPS library are rated across three emotive measures: pleasure (pleasant to unpleasant), arousal (calm to excited), and dominance (controlled to in-control). Football-related images were chosen using three pilot focus group sessions composed of members who self-reported their status as both fans and non-fans of American football (*N* = 44; 21 Fans and 23 Non-Fans). These focus groups independently rated a subset of IAPS images' pleasure (*M* = 7.6, *SD* = 1.3), arousal (*M* = 7.5, *SD* = 2.1), and dominance ratings (*M* = 7.1, *SD* = 2.0). These mean ratings fell within 0.5 points of the original Lang et al. (25) normative ratings, suggesting that the groups' ratings were reliable. These focus groups then rated 32 football-related images using the same measures and methods to record pleasure (*M* = 6.1, *SD* = 1.9), arousal (*M* = 5.8, *SD* = 2.2), and dominance (*M* = 5.8, *SD* = 1.7). The three focus groups did not rate the two image types (general violence and football violence) differently, as confirmed by the lack of an interaction between focus groups and image type in an omnibus three-way ANOVA (*F*_(2, 88)_ = 0.95, *p* = 0.39). In addition, fans and non-fans also did not rate the stimuli differently across the three focus groups, as confirmed by the lack of an interaction between focus groups and fan identity in an omnibus three-way ANOVA (*F*_(2, 88)_ = 0.94, *p* = 0.39). The interaction between all three factors (fan type, image type and focus group) was also non-significant for the ratings (3-way ANOVA, *F*_(2, 88)_ = 0.24, *p* = 0.78). The images from this image set were then used as stimuli in the fMRI experiment.

### Procedure

**Figure 1:**
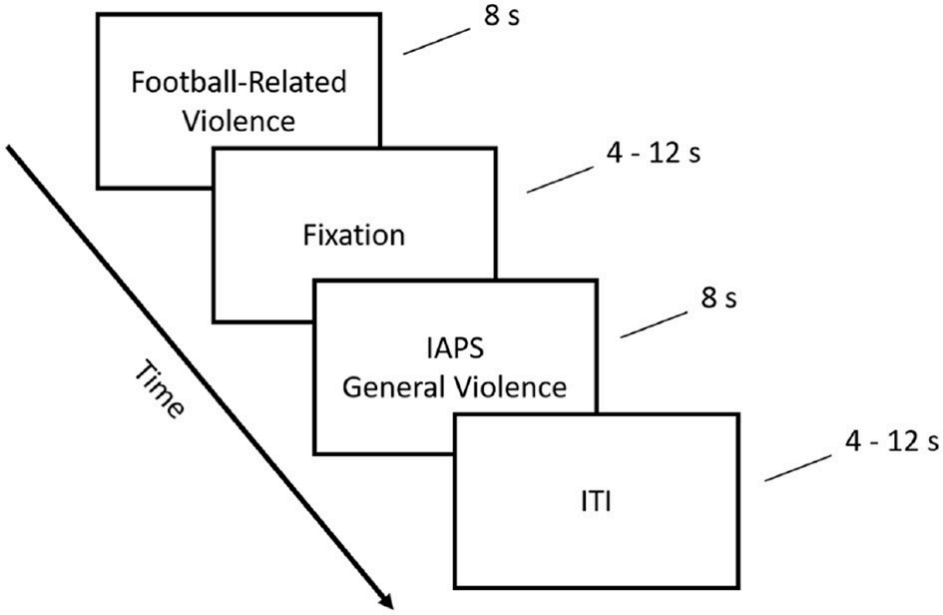
Viewing task. The experimental procedure used by all participants while in the MRI scanner.

After participants were consented and briefed, they prepared for their scans by changing into surgical scrubs. Researchers then verbally instructed to the participants what they would be experiencing while in the MRI scanner. Figure 1 illustrates the sequence in which different stimuli were presented in a given trial. Accordingly, participants viewed an image of football-related violence for 8 s followed by a fixation cue between 4 and 12 s, an IAPS image of general violence for another 8 s, and an inter-trial interval (ITI) between 4 and 12 s in length. Participants experienced this sequence a total 30 times (i.e., 30 trials). Images were randomized between participants, and pseudorandom ITI lengths were optimized using Optseq (http://surfer.nmr.mgh.harvard.edu/optseq/). Participants were then debriefed and monetarily compensated for their time.

### Data acquisition

Functional and anatomical data were acquired using a 7T Siemens (Erlangen, Germany) MAGNETOM scanner equipped with a 32-channel head coil (Nova Medical, Wilmington, MA). Functional images were acquired using a gradient echo, multiband echo planar imaging (EPI) sequence (26) with the following parameters: 45 slices acquired parallel to the AC-PC (anterior commissure – posterior commissure) line, voxel size: 2 × 2 × 2 mm^3^, TR (repetition time)/TE (echo time): 1,000/20 ms, 70° flip angle, FOV (field of view) = 200 mm, base/phase resolution of 96/100%, the anterior-to-posterior phase encoding direction, multiband (MB) slice acceleration factor of 3, partial Fourier of 6/8, interleaved acquisition, 420 measurements.

The whole-brain high resolution three-dimensional (3D) magnetization-prepared rapid gradient echo (MPRAGE) sequence was used to acquire anatomical data with the following parameters: 240 slices, voxel size: 1.2 × 1.2 × 1.2 mm^3^, TR/TE: 1,900/2.75 ms, 7° flip angle, base/phase resolution: 192/100%, in-plane phase-encode acceleration factor (iPAT) GRAPPA acceleration factor of 2, FOV read/ phase: 224 mm/100%, bandwidth: 240 Hz/Px, ascending acquisition.

### Data pre-processing and analysis

Results were computed using the MATLAB-based Statistical Parametric Mapping (SPM12) toolbox (http://www.fil.ion.ucl.ac.uk/spm/software/spm12/). Pre-processing steps were followed according to Poldrack et al. (27) and included slice-timing correction, realignment, co-registration, normalization to MNI (Montreal Neurological Institute) coordinates, and spatial smoothing. Realignment (i.e., motion correction) minimized least-squares errors along 6 degrees of freedom (3 rotations, 3 translations). All images with head movement (framewise displacement) above 0.2 mm were replaced by images derived using the cubic spline temporal interpolation to ensure continuous temporal data for all subjects. All mean functional images created during alignment were then co-registered with individual anatomical images obtained via MPRAGE. Remaining functional images were re-sliced to align with this anatomical image as a reference. Spatial normalization was used to nonlinearly warp all participants' brains to the MNI template image. To increase the signal-to-noise ratio after pre-processing, data were smoothed using a Gaussian kernel with a full-width half-maximum of 8 × 8 × 8 mm.

General Linear Modeling (GLM) was used to estimate the extent of activation of voxels in the brains of individual participants toward both general and football-related violence. In addition to regressors corresponding to the above two conditions, other nuisance regressors based on head movement and derivatives of the hemodynamic response function were used in the design matrix of the GLM in order to reduce noise in the data. This first-level analyses provided contrast images containing responses to football-related violence vs. general violence at the individual subject level. These contrasts were later entered into a second-level analysis (*t*-test) where individual subject-level activations were compared across groups (i.e., Fan vs. Non-Fans) to determine brain regions whose activation differed significantly between fans and non-fans.

## Results

**Figure 2:**
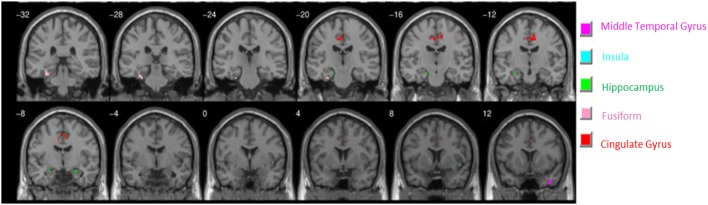
Non-Fans > Fans Contrast. Cluster activation for the contrast of the Non-Fan > Fan groups across all conditions. Non-fans reliably show more activation in key areas of the brain compared to fans.

In our task involving viewing football and general violence related images (Figure 1), individuals that identified themselves as North American football Fans exhibited decreased activation (to both types of violence taken together) in multiple regions of the brain when compared to Non-Fans (Figure 2). A between-groups contrast of Non-Fans versus Fans confirms these regions of interest (ROIs) with a FDR corrected *p*-value threshold of 0.05. These ROIs (Table 1 shows the regression coefficients for each of the four conditions and the *t*-values for the comparison between Fans and Non-Fans) included the bilateral hippocampus, right Insula, left fusiform gyrus, bilateral cingulate gyrus, and right middle temporal gyrus. Many of these areas have been implicated in emotion regulation, the perception of others' pain (i.e., empathy), and the neural origin of violent behaviors (28). Of particular interest is that the Fan group showed less activation in these areas, which may mark a decreased empathetic response that often comes with increased exposures to violence.

**Table 1:** Contrast results.

**Region (AAL)**	**MNI coordinate (x, y, z)**			**Volume (mm3)**	***t*-value**	**β Fans football violence**	**β Fans general violence**	**β non-fans football violence**	**β non-fans general violence**
Right hippocampus	28	−10	−20	20	5.90	0.14 ± 0.06	0.26 ± 0.08	0.28 ± 0.08	0.24 ± 0.06
Left hippocampus	−26	−12	−22	14					
Right insula	46	6	−6	10	8.27	0.2 ± 0.2	0.4 ± 0.18	1.1 ± 0.45	1.0 ± 0.42
Left fusiform gyrus	−28	−28	−28	123	17.21	0.1 ± 0.06	0.26 ± 0.07	0.24 ± 0.21	0.3 ± 0.19
Left cingulate gyrus	−8	−18	40	54	7.07	0.28 ± 0.13	0.4 ± 0.15	0.8 ± 0.2	0.81 ± 0.17
Right cingulate gyrus	8	−14	42	78					
Right middle temporal gyrus	40	10	−42	40	14.92	0.08 ± 0.045	0.15 ± 0.046	0.22 ± 0.06	0.18 ± 0.03

*Brain regions (along with their MNI coordinates and volume) which showed significantly (p < 0.05, FDR-corrected) lower response to football violence as compared to general violence in the Fan group. The regression coefficients (β) for each of the four conditions (football and general violence in Fans and Non-fans) are also provided for all ROIs. It is noteworthy, that in all ROIs, except bilateral hippocampus, Fans had significantly (p < 0.05, FDR-corrected) lower response to violence (football and general violence taken together) as compared to Non-fans (as shown in Figure 2). T-values obtained from a between groups t-test between Fans and Non-fans (irrespective of the type of violence) is also provided*.

**Figure 3:**
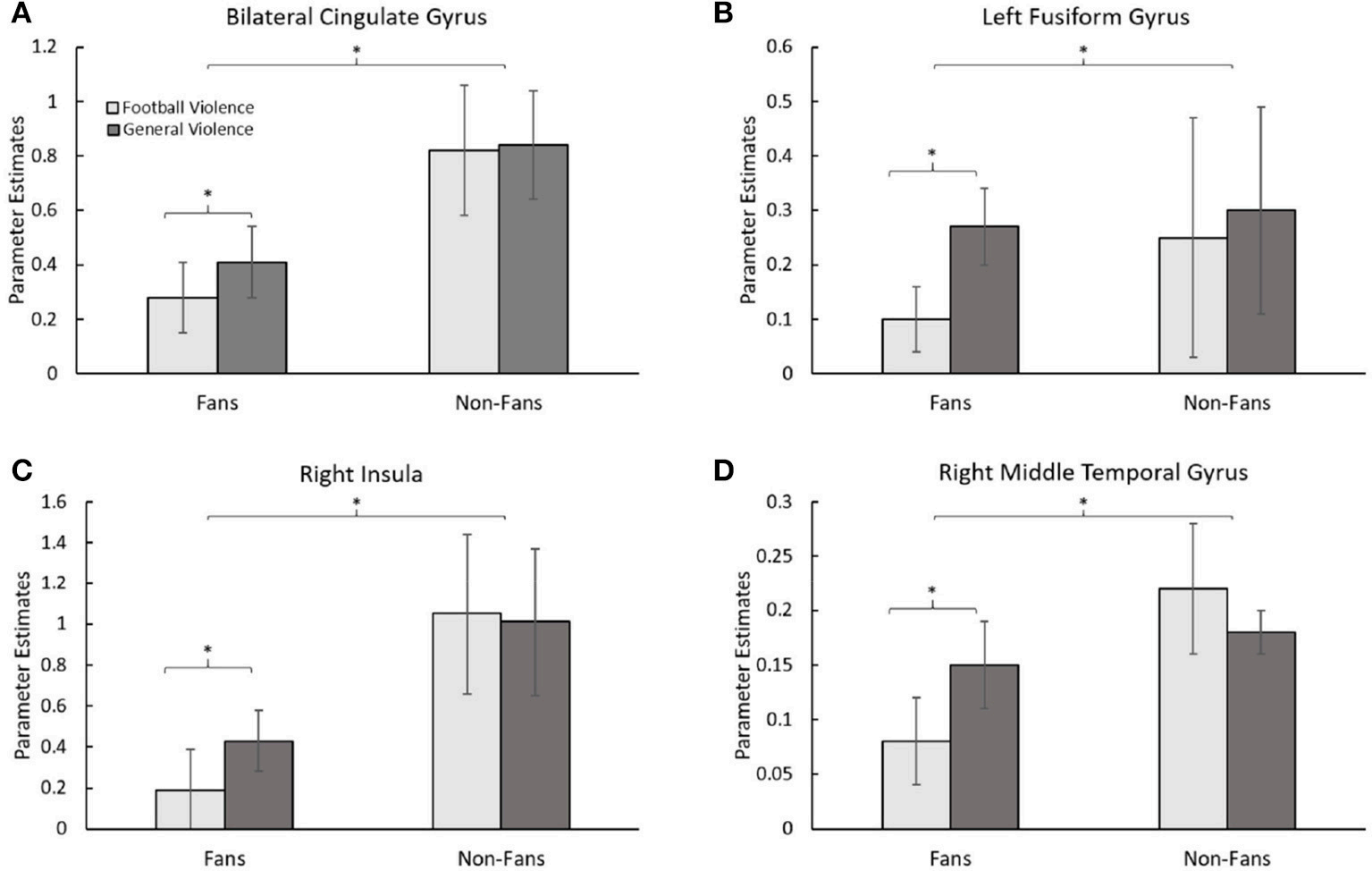
Functional differences in Fans vs. Non-Fans. Parameter estimates of ROI activation for football-related violence and general violence across fan classification. **(A)** shows a comparison of the activation in the bilateral cingulate gyrus. **(B)** shows a comparison of the activation in the left fusiform gyrus. **(C)** shows a comparison of the activation in the right insular cortex. **(D)** shows a comparison of the activation in the right middle temporal gyrus. For all the four regions, the Fan group showed significantly (*p* < 0.05, FDR corrected) less activation for football violence as compared to general violence. Also, all regions showed significantly (*p* < 0.05, FDR corrected) less activation in the Fan, as compared to the Non-Fan group when violence type was combined. The asterisk represents a difference of *p* < .05 between the comparisons.

In order to determine effects specific to football violence in fans, separate two-way ANOVAs were performed with groups (Fan vs. Non-Fan) and condition (sports violence vs. general violence) as factors for each brain region in Table 1. Except for the bilateral hippocampus, all other regions showed effects specific to football violence in the Fan group. Accordingly, when fans viewed images of football-related violence, these areas showed less activation (*p* < 0.05, FDR corrected) compared to when fans viewed images of general violence. These areas, the bilateral cingulate gyrus (Figure 3A), the left fusiform gyrus (Figure 3B), the right insula (Figure 3C), and the right middle temporal gyrus (Figure 3D), reflect a difference in affective processing when comparing sports-related and general violence images. Non-fans, however, showed no such effect. When the Non-Fan group viewed both sports violence and general violence, these areas show no difference in activation. However, the Non-Fan group had greater activity (*p* < 0.05, FDR corrected) in these regions compared to the Fan group when they were compared with both violence types combined. It is noteworthy that these brain regions are integral to the perception of pain and violence toward other individuals (29).

## Discussion

Our cross-sectional experiment demonstrates a neurofunctional relationship between violence exposure and North American football fans. It should be noted that the causal directionality of this relationship could not be discerned due to the cross-sectional nature of the current study. Nevertheless, these findings suggest that how individuals perceive sports interactions may be fundamentally different based on their self-identification as a fan or non-fan of the sport. Even though fans and non-fans did not rate images of sports violence and general violence differently (as determined by the lack of an interaction of fan identity and image type in an omnibus three-way ANOVA, *F* = 0.19, *p* = 0.68), they show profound neurofunctional differences based on their group classification. Areas responsible for perceiving pain in others showed less activation in fans when viewing football-related violence, such as a tackle or an aggressive collision between two people. Non-fans, however, perceived this football-related violence much the same as non-sports-related violence, such as an assault or robbery, with these same areas showing nondifferential activation across violence type. Extrapolating these results, a fan of wrestling may not perceive a chokehold as an act of violence between two people, but a non-fan may perceive it as such. These findings support Anderson and Bushman's (1) General Aggression Model which predicts increased exposure to violence (sports-related or not) should reduce activation in neural regions responsible for empathetic responses (4).

Decreased activity in the cingulate has been observed in individuals with a higher probability of participating in violent or impulsive behaviors (28, 30). In the present study, this area may be active because of its role in violent behavior, but the cingulate has also been found to be active when perceiving pain (31) and the pain of others (29). Non-fans, then, may perceive both sports violence and general violence as being equally painful while fans may perceive all violence to be relatively less painful and specifically football-related violence to be less painful than general violence. Like the cingulate, the insula has also been implicated in the perception of pain toward the self and toward others (29). The insula has also been shown to be active when threatening images (such as angry faces) are presented (32, 33). Therefore, fans of North American football having less insular activation may be because images of sports violence are not perceived to be threatening by them, whereas images of general violence were, even though both types of images were rated similarly by them.

The middle temporal gyrus and specifically the temporal pole is an area responsible for mediating visual and emotional information (34). This area may be critical for facilitating empathy and relating to others (34, 35). Fans, then, may “feel for” recipients of general violence compared to recipients of sports violence. The fusiform gyrus, however, has long been implicated in its role in face perception (36). It is possible that fans perceive the individuals in the sports violence images not as people but as players, or an individual part of a greater whole. Non-fans, however, may see these individuals much like themselves and are able to take their perspective in the action. Within the Fan group, the hippocampus also showed less activation for violence in general compared to non-fans. Activation in the hippocampus was, however, not significantly different for the interaction between fan status and type of violence.

Our results are distinct from those of emotional appraisal or regulation. The orbital frontal cortex and the amygdala, often associated with emotion regulation (28, 37) did not show significantly different activations for either violence type or between fan groups. One might expect a difference in amygdala activation for sports violence and general violence between fans and non-fans, where fans show less neural response toward football-specific stimuli. These areas are likely not active, nor different between groups, because our task did not involve cognitive appraisal of emotional stimuli.

Like many other studies focusing on violence and media (38, 39), the causal relationship is uncertain. It is possible that individuals become fans of contact sports such as North American football because they do not perceive recipients of tackles or collisions to suffer any pain. However, it may be just as possible that fans of football show less activation in areas responsible for perceiving pain in others because of the repeated exposure to football-related violence. Likewise, non-fans may be discouraged from football because they perceive players to be subjected to painful treatment, or perhaps their empathetic neural response is due to their infrequent exposure to football. Further research, including longitudinal experimental designs, is required to establish the causal mechanisms that lead to the differences in neurofunctional differences between fans and non-fans and how those factors change over time. We outline asepcts that can be investigated in such a future longitudinal investigation as follows:

First, a concern raised by the current experiment for all levels of North American football is the ability of spectators to recognize the violent aspects of North American football and react to that violence in a way similar to those that have not been desensitized. As discussed above, whether this is actually desensitization or not can be investigated in future longitudinal experiments.

Second, if it is indeed desensitization due to context exposure to violence in the sport, one needs to investigate the possibility that this same type of desensitization is also occurring within coaches, trainers, and the medical staff who are collectively responsible for player health and safety. Players are largely considered to be the worst protectors of their own health; especially when it comes to the most competitive levels of North American football. According to the NFL Players' Association, the average career length of a professional football players is only 3.3 years, which leaves very little time for a player to realize their professional and monetary goals. With that in mind, it is not hard to understand why players often feel that they have no other choice than to play through the pain and are also reluctant to self-identify as having incurred an injury, especially of the type that requires removal from play (40). The combination of a players' desire to stay on the field at all costs and the desensitization of other personal may be leading to a dangerous environment for the long-term health and safety of the players, at all levels (Professional, Collegiate, and Youth). These factors are particularly troubling from a public health standpoint when one considers that a little over 1.1 million high school students played on their high school football team during the 2015–2016 school year alone according to the National Federation of State High School Associations, while an additional 1.23 million also played youth football (ages 6–12) in 2015 according to the latest data from the Sports & Fitness Industry Association (41). The potential impact of these many young people participating in North American football and possibly being exposed to the long-lasting injuries associated with repetitive head injuries is concerning. Future research should investigate this potential issue further, with an eye toward measures that avoid self-reported data collection (surveys and qualitative measures) and instead focus on the ability of the participant to identify and then react to on the field violence.

Third, longitudinal studies using larger samples aimed at understanding the neural mechanisms of fans' perception of violence in North American football is a key to better-informed policies aimed at rule changes in the sport. There is a distinct possibility that fans may become less loyal to North American football if additional rule changes are implemented which increase player safety and decrease the overall violence associated with the sport. Previous researchers have noted that on the field violence, especially unscripted on the field violence, is the most enjoyable part of the viewing process for fans (8–10). While recent rule changes to North American football to decrease the incidents of concussions have been met with only marginal resistance (7) from the fan perspective (for example, the so-called Targeting rule), it is an open question as to how far these types of rules can go before fans start to become less loyal. This issue is something that all stakeholders of North American football must be keen to avoid, and stakeholders could certainly look to previous rule changes in other sports that are deemed violent, to find examples of success and failure. Future research should examine the way that fans' enjoyment has been effected by rule changes, what rules fans find most important, and what it is about changes to rules that so negatively impact the experience of some fans.

Fourth, further investigation is also required to understand the purported phenomenon of desensitization to violence is specific to North American football, or if it extends to all contact sports as well, potentially increasing the public health threat associated with desensitization to violence. That said, the prior literature suggests that violence in sport may have a broader socio-cultural underpinning based on research performed on soccer hooliganism (42–44). These studies contend that fans of soccer may perceive acts of roughness not as violent acts against other individuals, but as a basic characteristic of a sport. Accordingly, because our results suggest that areas related to empathy (cingulate, insula, temporal lobe) are less active for fans, these hooligans may temporarily lose some ability to relate to one another when exposed to these sports events. Given what is known about other forms of media violence, this exposure to violence and aggression may also cause others to be more prone to acts of violence (38).

Fifth, future studies may probe the generalizability of the current findings to not just other sports, but all violent behaviors. Individual's perception of pain and violence have far-reaching implications for behavior in general. Aside from the aggressive, impulsive behaviors that may result in destructive acts like violence, the muted perception of pain and violence may increase suicidal risk factors, such as acquired capability (45). Game-related violence may change individuals' fearlessness about death, leading to higher likelihoods of suicidal ideation in individuals who are already predisposed toward it (46). In accordance with our findings, it may be possible that frequent exposure to contact sports may “numb” individuals to pain. This is particularly dangerous when considering individuals that are at high risk of suicidal ideation.

Certain limitations must be noted while interpreting the findings of this study. Most critically, the current study did not measure behavioral responses. For example, emotive measures could have been recorded within the scanner, with participants responding on how pleasurable, arousing, or dominating the current images are. This self-report data could be used in a parametric analysis to determine if these ratings correlate with neurofunctional differences. Additionally, the small sample size (*N* = 15) may limit the power of the statistical analysis, and future studies should seek to incorporate larger samples within Fan and Non-Fan groups, as well as examining the same participants in a longitudinal manner. While sample sizes of this nature are generally acceptable in fMRI studies (47), larger samples generally offer more impactful results. Finally, our basis on forming the Fan and Non-Fan groups were dependent upon participants' self-reporting the level of their fanhood. More objective, empirical methods should be employed in the future to ensure that participants' behaviors are reflective of their group.

In conclusion, the current study shows neurofunctional differences between fans and non-fans of North American football. Key areas of the brain respond differently when viewing violence, and for fans, these areas responded less to violence in the context of football. This finding does not demonstrate that football enthusiasts are more prone to violence or less sensitive to violent imagery, but instead, that violence within the context of football may provide less affective arousal compared to general violence.

## Author contributions

GD, KT, and DM designed the study. GD and DM obtained funding for the study. KT, YW, DM, and GD acquired the data. YW, KT, TD, and GD analyzed the data. GD, JK, TD, KT, and DM interpreted the results. All authors wrote the manuscript.

### Conflict of interest statement

The authors declare that the research was conducted in the absence of any commercial or financial relationships that could be construed as a potential conflict of interest.
